# Early Life Stress Influences Oxidative Stress Enzyme Activities in Liver, Heart, Kidney, Suprarenal Glands, and Pancreas in Male and Female Rat Pups

**DOI:** 10.3390/antiox13070802

**Published:** 2024-07-02

**Authors:** Bertha Fenton Navarro, Alexis Abraham Casimiro Aguayo, Yayr Luis Torres Gómez, Miguel Cervantes Alfaro, Luz Torner

**Affiliations:** 1 Laboratorio de Glicobiología y Farmacognosia, Facultad de Ciencias Médicas y Biológicas “Dr. Ignacio Chávez”, Universidad Michoacana de San Nicolás de Hidalgo. Av. Dr. Rafael Carrillo S/N, Esq. Dr. Salvador González Herrejón Bosque, Cuauhtémoc, Morelia 58020, Michoacán, Mexico; 1342163h@umich.mx (A.A.C.A.); 1635167b@umich.mx (Y.L.T.G.); 2 Laboratorio de Neurociencias, Facultad de Ciencias Médicas y Biológicas “Dr. Ignacio Chávez”, Universidad Michoacana de San Nicolás de Hidalgo, Morelia 58000, Michoacán, Mexico; miguel.cervantes@umich.mx; 3 Laboratorio de Neuroendocrinología, Centro de Investigación Biomédica de Michoacán, Instituto Mexicano del Seguro Social; Cam. de La Arboleda # 300, La Huerta, Morelia 58341, Michoacán, Mexico

**Keywords:** oxidative stress, maternal separation, antioxidant enzymes, organs, rat

## Abstract

Early life stress (ELS) is a risk factor for the development of chronic diseases resulting from functional alterations of organs in the cardiorespiratory and renal systems. This work studied the changes in oxidative stress enzyme activities (EAs) of SOD, CAT, GPX, GR, GST, NOS, MDA, and FRAP in different organs (heart, liver, kidney, adrenal glands (AGs), and pancreas) of male and female Sprague–Dawley rat pups on postnatal day (PN) 15, immediately after basal and acute or chronic stress conditions were accomplished, as follows: basal control (BC; undisturbed maternal pups care), stress control (SC; 3 h maternal separation on PN15), basal maternal separation (BMS; daily 3 h maternal separation on PN 1-14), and stress maternal separation (SMS; daily 3 h maternal separation on PN 1-14 and 3 h maternal separation on PN15). Acute or long-term stress resulted in overall oxidative stress, increase in EA, and reduced antioxidant capacity in these organs. Some different response patterns, due to precedent SMS, were observed in specific organs, especially in the AGs. Acute stress exposure increases the EA, but chronic stress generates a response in the antioxidant system in some of the organs studied and is damped in response to a further challenge.

## 1. Introduction

The Developmental Origins of Health and Disease (DOHaD) hypothesizes that environmental insults during childhood cause the development of chronic disease in adulthood. Emerging epidemiological data strongly support the idea that early life stress (ELS) caused by exposure to adverse childhood experiences is an independent risk factor for the development of chronic disease, being capable of predicting future cardiovascular, respiratory, and renal diseases; diabetes; and cancer [1,2].

Stress induces an increase in corticosterone secretion in rats, which activates glucocorticoid receptors (GRs). GRs modulate transcriptional factors in the nucleus, which increase mitochondrial membrane potential and mitochondrial oxidation in different cell types present in various organs [3]. This mechanism increases the cellular metabolic rate, promoting ATP synthesis and spontaneous superoxide. Free radicals, or reactive oxygen species (ROS) and reactive nitrogen species (RNS) [4], are molecules produced mainly by the mitochondrial electron transport chain. However, they are also formed as byproducts of several cellular enzymes, including NADPH oxidase and nitric oxide synthase oxidase (NOX), xanthine oxidase, and cytochromes P450, as well as by the activation of the immune system, lipid peroxidation, ischemia, or trauma [5]. In excess, they promote significant damage to biological macromolecules, such as DNA, protein, and lipids, which can promote cell death [6].

The defense mechanisms against oxidative stress include a cascade of antioxidant enzymes: first, the superoxide dismutases (cytosolic copper-zinc superoxide dismutase (CuZnSOD) [7] and mitochondrial manganese superoxide dismutase (MnSOD) catalyze the dismutation of the superoxide anion (O_2_^−^) to oxygen and hydrogen peroxide (H_2_O_2_), which is further detoxified by the enzymes catalase (CAT) and glutathione peroxidase (GPX), and CAT reduces H_2_O_2_ into molecular oxygen and water [8]. GPX performs a reduction of H_2_O_2_ to water and a reduction of organic hydroperoxides to their corresponding alcohols in the presence of glutathione (GSH), which is oxidized to glutathione disulfide (GSSG) [9]. The reduction of GSSG back to GSH is catalyzed by glutathione reductase (GR) using reduced nicotinamide adenine dinucleotide phosphate (NADPH) [10,11]. It has been shown that chronic stress may affect GSH levels [12,13]. In addition, some psychiatric disorders are characterized by GSH depletion [14], and the NADPH oxidase (NOX) family has also been implicated in psychiatric disorders [15].

Besides ROS, the exposure of organisms to stressors may lead to the overproduction of nitric oxide (NO) [16] and the nitrosylation of antioxidant enzymes [17]. The concentration of NO within biological systems is regulated by the activity of nitric oxide synthase (NOS) isoforms: NOS enzymes are widely distributed within mammalian tissues, such as the heart, brain, lung, endothelial cells, pancreas, kidney, and adrenal glands [18,19,20,21].

This work aimed to determine the simultaneous antioxidant enzyme profiles of SOD, CAT, GPX, GT, GR, NOS, malondialdehyde (MDA), and ferric-reducing ability potential (FRAP) in the heart, liver, kidneys, adrenal glands, and pancreas in male and female pups in response to acute or chronic stress (daily maternal separation). To the best of our knowledge, this is the first report that includes all enzymes in the organs mentioned above in male and female pup rats under basal and stress conditions.

## 2. Materials and Methods

### 2.1. Animals

We used 122 pups (male and female) from 16 litters of Sprague–Dawley dams. They were kept in animal facility rooms at a standard temperature (22–24 °C) with a 12 h light/dark cycle (7:00 a.m. on/7:00 p.m. off), and they had free access to food and water. The experimental procedures were approved by the Institutional Animal Care Committee UMSNH (CIC-2023, 2024-BFN) and the Instituto Mexicano del Seguro Social National Scientific Committee (R-2019-1602-015), according to official regulations for the use and care of laboratory animals in Mexico [22]. Further, the experimental protocols were accomplished following the rules and guidelines of the U.S. National Institutes of Health. Adequate measures were taken to minimize animal pain or discomfort and to reduce the number of animals used in the experiments.

### 2.2. Experimental Scheme

The dams were housed in a room separated from the rest of the colony. The day of delivery was considered postnatal day (PN) 0. All the pups were pooled and randomly distributed among the dams. The litters were culled to 8 to 10 pups (5 males and 5 females on PN1 where possible), and both the mothers and pups remained undisturbed unless their specific participation in experimental procedures was required. MS procedures started (9:00 a.m.) on PN1 and continued until PN14. Half of the litters (MS groups) were separated daily from the mother’s nest for 3 h and placed individually in small boxes with fresh bedding and a controlled temperature (32–35 °C) to prevent tactile contact with their siblings [23]. The other litters remained undisturbed in the mother’s nest (control groups). All litters received the usual care from the animal facilities, including cage cleaning without disturbing the nest.

**Figure 1:**
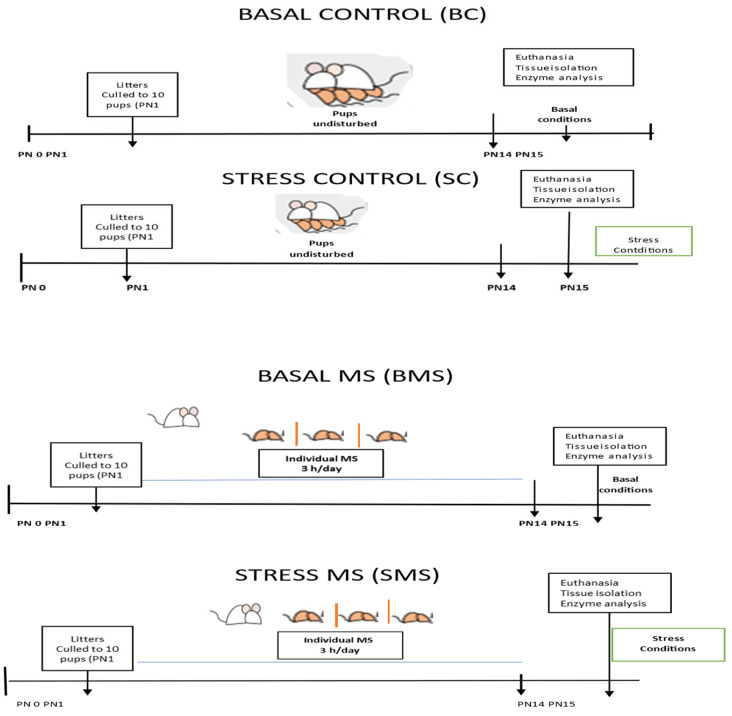
Experimental scheme of the animal groups.

Euthanasia conditions: On PN15, half of the groups were euthanized using pentobarbital (sedalphorte at 100 mg/kg of body weight; Salud y Bienestar Animal, Mexico, NOM-062-1999 [22]), with as little disturbance as possible (basal conditions), and the other half of the groups were subjected to acute stress (3 h MS) and euthanized at the end of this period (acute stress conditions) (Figure 1). The liver, heart, kidneys, adrenal glands, and pancreas were dissected and stored at −70 °C until analysis.

### 2.3. Tissue Homogenate Preparation

The frozen tissues were thawed in ice-cold 50 mM phosphate buffer pH 7.0 with an inhibitory protease cocktail (cOmplete™, Mini Protease Inhibitor Cocktail (Roche Rotkreuz, Switzerland)); the volume for the pancreas and adrenal glands was 200 µL, and, for the liver, kidney, and heart, the volume was 500 µL.

The tissues were subjected to three sonication cycles of 10 pulses on ice with a 1 min interval between cycles in a SONIC Vibra-Cell Model CV18. The resultant homogenate tissues were centrifuged at 14,000 rpm (SORVA LEGEND MACH 1.6R) (10537 G) for 20 min. The supernatant was placed in a new Eppendorf tube and stored at −30 °C until analysis.

The absorbances were measured using a Biotek Synergy HT plate reader (Agilent Technologies. Santa Clara, CA, USA).

### 2.4. Protein Concentration

Bradford reagent (Bio-Rad, Hercules, CA, USA) was bought and ready to use. A stock solution of bovine serum albumin in distilled water (10 mg/mL) was prepared as a protein standard. A dilution of bovine serum albumin was conducted for the measurements, starting with 1 mg/mL. The protein concentration is reported as mg/mL [24,25].

### 2.5. Superoxide Dismutase (SOD) Activity

The determination of SOD activity was carried out using a modification of the method described by Beyer and Fridovich 1987 and Ewing and Janero, 1995 [26,27]. The method is based on the ability of SOD to inhibit the reduction of the yellow compound NTB (nitrobluetetrazolium), which, in the presence of the superoxide radical (O_2_^−^), is reduced. This results in a compound with an intense blue color, formazan blue, which is generated by the hydroxylamine in alkaline solutions. Finally, the reduction of NTB is measurable spectrophotometrically at 560 nm.

The activity of SOD was determined using the following formula: USOD = (V/v), where U is the units, and V is the absorbance in the absence or V presence of SOD. One unit of SOD is the amount of enzyme capable of inhibiting 50% of NBT. Values are reported as U_SOD_/mg protein.

### 2.6. Catalase (CAT)

Catalase activity is based on the conversion of H_2_O_2_ into water and oxygen. The reaction mixture contained 33 mM H_2_O_2_ in water and oxygen. The specific activity (SA) was calculated using the following formula: SA (µmol H_2_O_2_/mL/min) (ΔDO/min) (TV) (DF)/(SV) (min) (0.0436), where ΔDO is the absorbance of the sample, TV is the total volume, DF is the dilution factor, SV is the sample volume, and €H_2_O_2_ is 0.0436, which is the extinction coefficient of H_2_O_2_ [28]. The activity is reported as units of catalase/mg protein (U/mg protein).

### 2.7. Glutathione Peroxidase (GPX)

The technique is based on the oxidation of guaiacol in the presence of H_2_O_2_ by peroxidase activity, resulting in tetraguaiacol and H_2_O as a product. The reaction was initiated by the addition of H_2_O_2_ and monitored by measuring the increase in absorbance at 420 nm [29]. Peroxidase activity is expressed in nmol of the guaiacol test oxidized per min, using a molecular extinction coefficient of 26.6 mM^−1^ cm^−1^ for calculation [30]. The product is expressed as GPX (U/mg protein).

### 2.8. Glutathione Reductase (GR)

Glutathione reductase catalyzes the reduction of glutathione disulfide (GSSG) to the sulfhydryl form of reduced glutathione (GSH) [31]. Reduced glutathione reacts with the superoxide radical (O_2_^−^), increasing oxygen consumption and forming oxidized glutathione. The reduced glutathione level was measured in the tissues, as described by Sedlak and Lindsay (1968) [32], using 5,5′dithiobis (2-nitrobenzoic acid) reduced by SH groups to form 1 mole of 2-nitro-5-mercaptobenzoic acid per mole of SH. Methanol was added and measured at 412 nm. The product is expressed as GSH (µmol/mg protein) [31,32].

### 2.9. Glutathione S Transferase (GST)

The reaction was measured by conjugating 1-chloro, 2,4-dinitrobenzene (CDNB) with reduced glutathione (GSH). This was carried out by measuring the increase in absorbance at 340 nm. One unit of enzyme conjugates 10 nmol of CDNB with reduced glutathione per minute at 25 °C. GST activity = [(Abs 340/min)/0.0096 μM^−1^/cm] × (1.0 mL/0.1 mL) × any sample dilution = U/mL. The results are reported as GST (U/mL) [33].

### 2.10. Nitric Oxide Synthase (NOS)

Nitrate and nitrite are the stable degradation products of NO produced by NOS. Griess’s 1858 reaction marks the presence of organic nitrites. Nitrite is detected by the formation of a pink color in a sample containing NO_2_. When sulfanilic acid is added, the nitrites form a diazonium salt; when alpha-naphthylamine is added, a pink color develops. A sodium nitrite standard curve was used (0–25 µM). Problems were interpolated in the standard curve. The results are expressed as µmol nitrites/L [34].

### 2.11. Malondialdehyde

Malondialdehyde (MDA) results from the lipid peroxidation of polyunsaturated fatty acids. It was evaluated from a modification of Ohkawa et al. (1979) [35] and Rezaeizadeh A. (2011) [36], using butylated hydroxytoluene (BHT), sodium dodecyl sulfate (SDS), and thiobarbituric acid (TBA). It was incubated for 1 h at room temperature; then, the pink supernatant was placed in a new microplaque and measured at 532 nm. A standard curve was prepared with 2.5–50 μM/L concentrations of TEP (1,1,3,3-tetraethoxypropane). The results are expressed as nmol MDA/g tissue.

### 2.12. FRAP (Ferric-Reducing Ability Potential)

The ferric-reducing ability was performed using the method described by Benzie and Strain with modifications. This method is based on the reduction of a colorless ferric complex (Fe^3+^ tripyridyltriazine (TPTZ)) to a blue-colored ferrous complex (Fe^2+^ tripyridyltriazine) by the action of electron-donating antioxidants at low pH, and absorbance was monitored at 593 nm using a BioTek Synergy HT spectrophotometer (Agilent Technologies. Santa Clara, CA, USA). A standard curve was prepared using different concentrations (0–1000 µM) of FeSO_4_. Problem samples were interpolated, and the results are expressed as µmol FeSO_4_/mg protein [37,38].

### 2.13. Statistical Analysis

A statistical analysis was carried out using descriptive statistics with measures of central tendency and the dispersion of values (mean ± standard error). If the data distribution was normal, variance (ANOVA) (two-way ANOVA) was analyzed to determine the significant differences. A post hoc analysis was carried out using Tukey’s test. One symbol = *p* < 0.05, two symbols = *p* < 0.01, three symbols = *p* < 0.001, and four symbols = *p* < 0.0001. Factor 1 was the treatment of the groups. Factor 2 was the euthanasia protocol. Factor 3 was gender and the interaction of 1 × 2 factors. The sample size was 8 to 10 animals per group. The statistical results were compared: * comparison between basal control (BC) and stress control (SC); ° comparison between basal maternal separation (BMS) and basal control (BC); # comparison between basal maternal separation (BMS) and stress maternal separation (SMS).

## 3. Results

**Figure 2:**
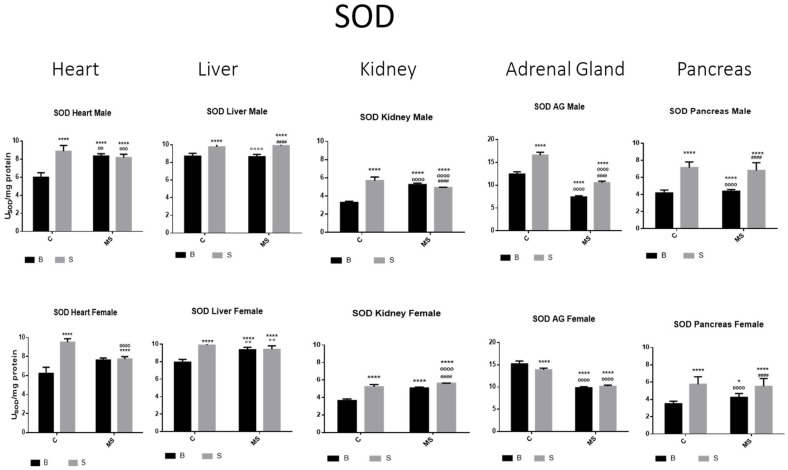
Total superoxide dismutase (SOD) enzyme activity. Male results are presented in the upper row, and female results are presented in the lower row. Groups: basal control (BC), stress control (SM), basal maternal separation (BMS), and stress maternal separation (SMS). * Differences from basal control; ° differences from stress control; and # comparison between BMS and SMS. One symbol = *p* < 0.05, two symbols = *p* < 0.01, three symbols = *p* < 0.001, and four symbols = *p* < 0.0001. Activity in SC was increased compared with BC in male and female organs. In the BMS group, activity was increased compared with BC, except for in AGs and pancreas. In the SMS group, activity was increased compared with BC in all male and female organs.

**Figure 3:**
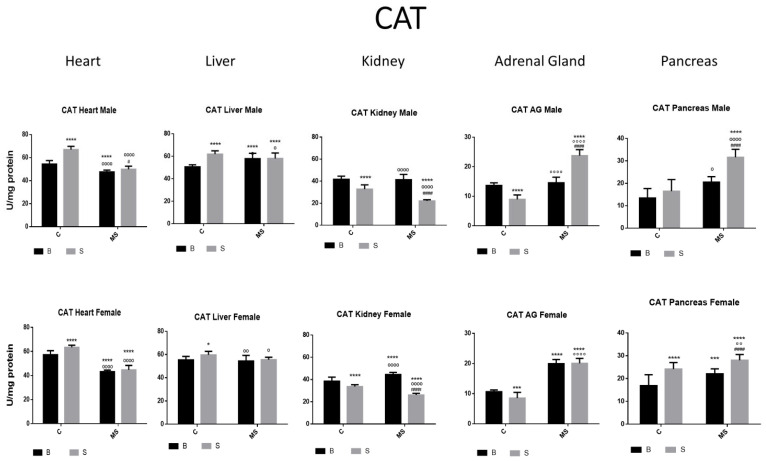
Catalase (CAT) enzyme activity. Male results are presented in the upper row, and female results are presented in the lower row. Groups: basal control (BC), stress control (SM), basal maternal separation (BMS), and stress maternal separation (SMS). * Differences from the basal control; ° differences from stress control; and # comparison between BMS and SMS. One symbol = *p* < 0.05, two symbols = *p* < 0.01, three symbols = *p* < 0.001, and four symbols = *p* < 0.0001. In SC, activity was increased in the heart, liver, and pancreas. In kidneys and AGs, activity was reduced when compared with BC. Activity in BMS groups was either increased, reduced, or without change depending on the group and gender. CAT activity in SMS groups was significantly increased in the heart, liver, AGs, and pancreas.

**Figure 4:**
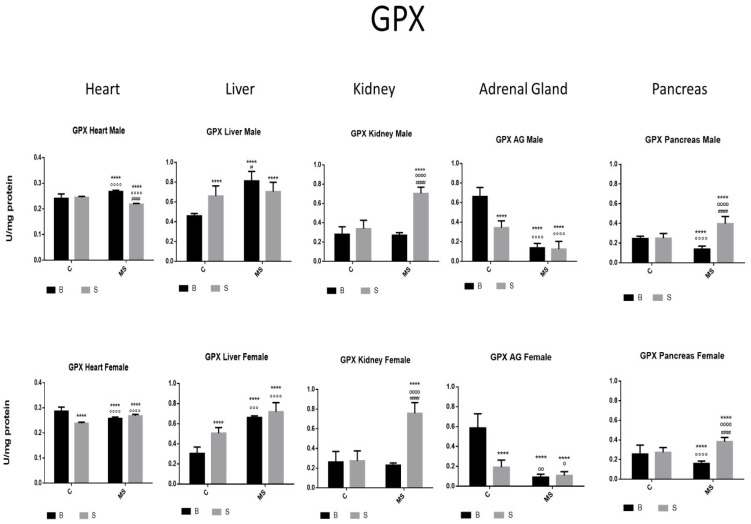
Glutathione peroxidase (GPX) enzyme activity. Male results are presented in the upper row, and female results are presented in the lower row. Groups: basal control (BC), stress control (SM), basal maternal separation (BMS), and stress maternal separation (SMS). * Differences from the basal control; ° differences from stress control; and # comparison between BMS and SMS. One symbol = *p* < 0.05, two symbols = *p* < 0.01, three symbols = *p* < 0.001, and four symbols = *p* < 0.0001. When compared with BC, SC showed increased activity in the liver; no change in activity was found in the heart, kidney, and pancreas; and reduced activity was found in AGs. The BMS group showed increased activity in the heart and liver and reduced activity in the kidney, AGs, and pancreas. SMS groups showed increased activity in the heart, kidney, and pancreas and reduced activity in the liver and AGs.

**Figure 5:**
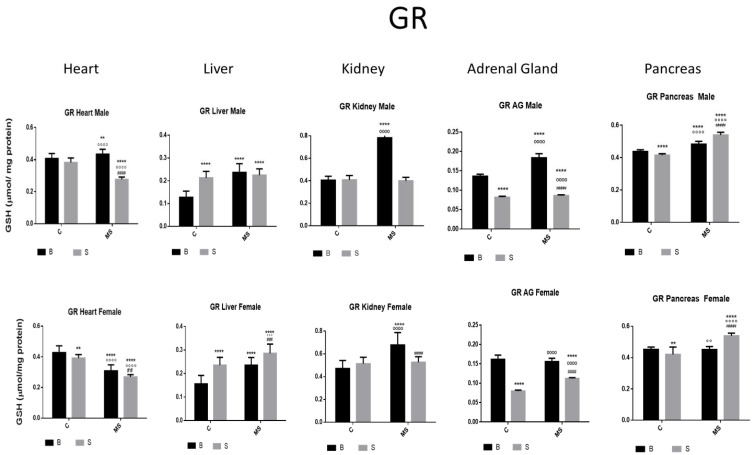
Glutathione reductase (GR) enzyme activity. Male results are presented in the upper row, and female results are presented in the lower row. Groups: Basal control (BC), stress control (SM), basal maternal separation (BMS), and stress maternal separation (SMS). * Differences from basal control; ° Differences from stress control, and # comparison between BMS and SMS. Two symbols = *p* < 0.01, three symbols = *p* < 0.001, and four symbols = *p* < 0.0001. Compared with BC, SC showed reduced activity in AGs and pancreas, and increased or no changes in the activity in the heart, liver, and kidney. BMS groups showed increased activity in all organs when compared with BC. In SMS groups, activity was increased in the liver and pancreas and was reduced in the heart, kidney, and AGs.

**Figure 6:**
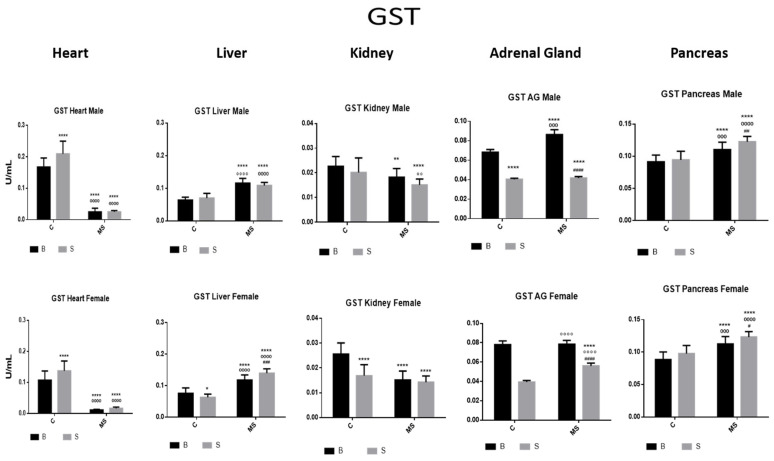
Glutathione S transferase (GST) enzyme activity. Male results are presented in the upper row, and female results are presented in the lower row. Groups: basal control (BC), stress control (SM), basal maternal separation (BMS), and stress maternal separation (SMS). * Differences with the basal control; ° differences from stress control; and # comparison between BMS and SMS. One symbol = *p* < 0.05, two symbols = *p* < 0.01, three symbols = *p* < 0.001, and four symbols = *p* < 0.0001. Compared with BC, SC showed increased activity in the heart, no changes in liver, kidney and pancreas, and significantly reduced activity in AGs. Compared with BC, the activity in BMS groups was reduced in the heart and kidney and increased in the liver, AGs, and pancreas. Compared with BC, SMS groups showed reduced activity in the heart, kidney, and AGs, and significantly increased activity in the liver and pancreas.

**Figure 7:**
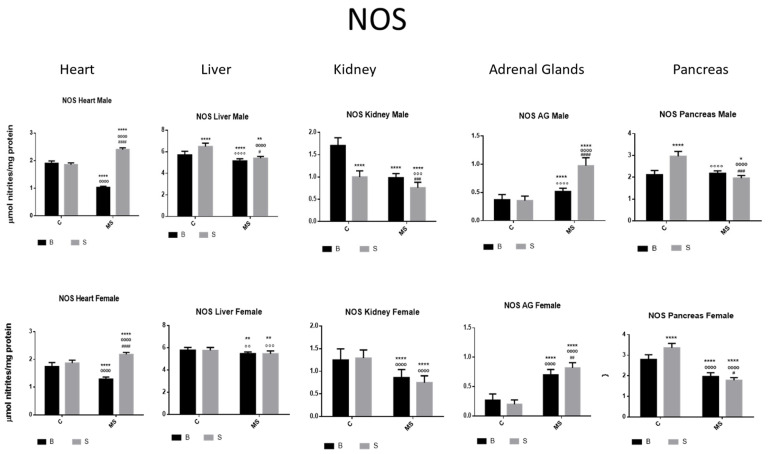
Nitric oxide synthase (NOS) enzyme activity. Male results are presented in the upper row, and female results are presented in the lower row. Groups: basal control (BC), stress control (SC), basal maternal separation (BMS), and stress maternal separation (SMS). * Differences from basal control, ° differences from stress control, and # comparison between BMS and SMS. One symbol = *p* < 0.05, two symbols = *p* < 0.01, three symbols = *p* < 0.001, and four symbols = *p* < 0.0001. Compared with BC, SC showed increased activity in liver and pancreas and reduced activity in kidney. Compared with SC, BMS groups showed reduced activity in heart, liver, kidney, and pancreas, and increased activity in AGs. Compared with BC, SMS groups showed increased activity in heart and AG and reduced activity in the other organs.

**Figure 8:**
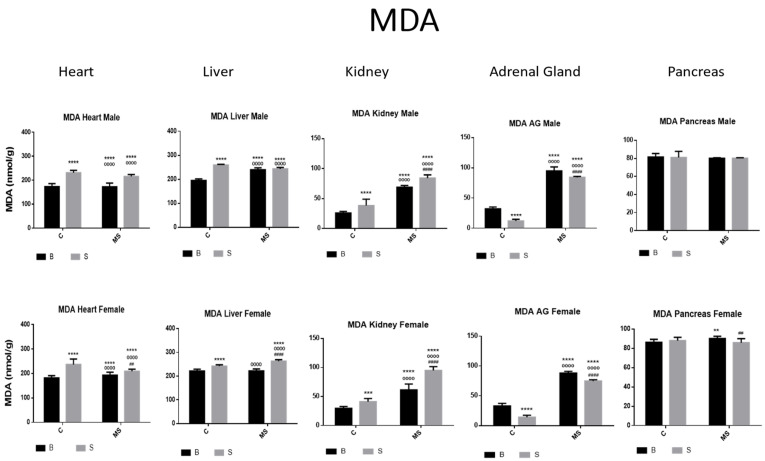
Malondialdehyde (MDA). Male results are presented in the upper row and female results are presented in the lower row. Groups: basal control (BC), stress control (SC), basal maternal separation (BMS), and stress maternal separation (SMS). * Differences from the basal control, ° differences from stress control, and # comparison between BMS and SMS. Two symbols = *p* < 0.01, three symbols = *p* < 0.001, and four symbols = *p* < 0.0001. MDA was found to be increased in heart, liver, and kidney and reduced in AGs when compared with BC. In BMS and SMS groups, activity was increased in all organs, except for pancreas, where no differences were found.

**Figure 9:**
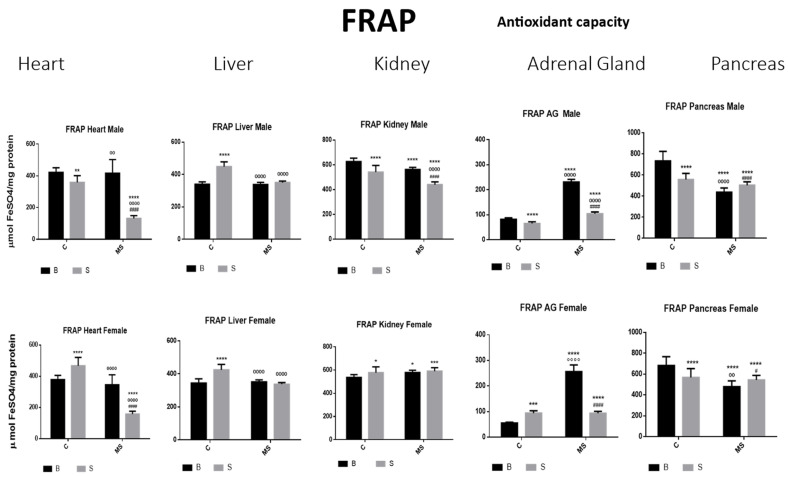
Ferric-reducing ability potential (FRAP). Male results are presented in the upper row and female results are presented in the lower row. Groups: basal control (BC), stress control (SC), basal maternal separation (BMS), and stress maternal separation (SMS). * Differences from the basal control (*), ° differences from stress control and # comparison between BMS and SMS. One symbol = *p* < 0.05, two symbols = *p* < 0.01, three symbols = *p* < 0.001, and four symbols = *p* < 0.0001. Compared with BC, the SC male groups showed a reduction in the amount of FRAP in heart, kidney, AGs, and pancreas; in liver, this was increased. In SC females, FRAP was increased in all organs, except for in the pancreas. In BMS groups, FRAP activity was reduced in the kidney and pancreas of males but increased in the kidney of females. In the male and female SMS groups, FRAP was significantly reduced in the heart, kidney, AGs, and pancreas. In the liver, no changes were observed between the BMS and SMS groups.

Figure 2, Figure 3, Figure 4, Figure 5, Figure 6, Figure 7, Figure 8 and Figure 9 depict the results obtained for the different tissues studied, namely the heart, liver, kidney, adrenal glands, and pancreas. The male and female rats were separated into the following groups: basal control (BC), stress control (SM), basal maternal separation (BMS), and stress maternal separation (SMS). The statistical results are presented in Table 1, Table 2, Table 3 and Table 4.

There were no statistical differences between the males and females in terms of the values obtained for all enzymes and all organs studied.

In the following section, the enzyme activities in specific organs are described under different experimental conditions, and no distinction is made between males and females because no statistical differences were found.

- Total Superoxide Dismutase (SOD) activity

SOD activity was increased in the stress control (SC) compared with basal control (BC) in all organs studied (the heart, liver, kidney, adrenal glands (AGs), and pancreas) in the male and female rats. In the maternal separation (BMS) groups, a significant increase in activity in the kidney was observed compared to the BC. In the BMS group, a reduction in the activity in the AGs and pancreas was found. The SMS groups presented an increase in enzyme activity in all organs in contrast with the BC. When compared with the BMS groups, we found increases in all organs, except for the heart, where the activity did not change (Figure 2 and Table 1A).

- Catalase (CAT) activity

In the SC group, CAT activity was increased in the heart, liver, and pancreas compared with the BC group. In the kidney and AGs, a reduction was found. CAT activity in the BMS groups was either increased, reduced, or without change depending on the group and gender. CAT activity in the SMS groups was significantly increased in the heart, liver, AG, and pancreas (Figure 3 and Table 1B).

- Glutathione peroxidase (GPX)

In the SC, BMS, and SMS groups, there was an increase or no change in the activity of the GPX enzyme in the heart, liver, and kidney compared with the BC of SC. In the SMS groups, we found a significant increase in GPX activity in the liver and pancreas compared with the BC, but this was reduced in the heart, kidney, and AGs. In the SMS groups, there was increased activity in the heart, kidney, and pancreas and reduced activity in the liver and AGs (Figure 4 and Table 2A).

- Glutathione Reductase (GR)

There was an increase or no change in GR activity in the heart, liver, and kidney of the SC groups compared with in the BC, while, in the AGs and pancreas, it was reduced. In the BMS groups, the activity was increased in all organs, while, in the SMS groups, the activity was increased in the liver and pancreas and reduced in the heart, kidney, and AGs (Figure 5 and Table 2B).

- Glutathione S Transferase (GST)

The SC showed increased GST activity in the heart and significantly reduced GST activity in the AGs compared with the BC. The BMS groups showed reduced GST activity in the heart and kidney, compared with the BC groups. In the SMS groups, there was a reduction in GST activity in the heart, kidney, and AGs, while, in the liver and pancreas, GST activity was increased (Figure 6 and Table 3A).

- Nitric Oxide Synthase (NOS)

NOS activity was increased in the liver and pancreas and reduced in the kidney of the SC groups compared with the BC groups. In the BMS groups, NOS activity was decreased in the heart, liver, kidney, and pancreas compared with in the BC groups; however, it was increased in the AGs. In the SMS groups, NOS activity was increased in the heart and AGs but was reduced in the other organs (Figure 7 and Table 3B).

- Malondialdehyde (MDA)

MDA activity was increased in the heart, liver, and kidneys and reduced in the AGs of the SC compared with the BC groups. Compared with BC and BS, the BMS and SMS groups showed increased MDA activity in all organs, except for in the pancreas, where no differences were found (Figure 8, and Table 4A).

- Ferric-Reducing Ability Potential (FRAP)

Compared with BC, there was a reduction in the amount of FRAP in the SC male groups in the heart, kidney, AG, and pancreas; in the liver, this was increased. In the SC females, FRAP was increased in all organs, except for in the pancreas. In the BMS groups, FRAP activity was reduced in the kidney and pancreas of the males but increased in the kidney of the females. In the male and female SMS groups, FRAP was significantly reduced in the heart, kidney AGs, and pancreas. In the liver, no changes were observed between the BMS and SMS groups (Figure 9 and Table 4B).

## 4. Discussion

Maternal separation (MS) results in disrupted maternal care, and the proximity of siblings contributes to brain development during the sensitive period. MS decreases the number of inhibitory neurons and synapses and causes an excitatory and inhibitory imbalance in the medial prefrontal cortex and hippocampus, which are regions involved in the negative control of the HPA axis. A mother’s contact regulates serotonergic system activity through 5-HT2 receptors in key prefrontal regions during early life (10- to 12-year-old rat pups). Thus, maternal care affects the adaptive/maladaptive development of brain circuits implicated in adult pathology. Among other things, it modulates the release of stress hormones, changes ultrasonic vocalizations (USVs), and provides olfactory tactile inputs to pups [39].

The uninterrupted activation of the HPA axis induces oxidative stress [3,40], characterized by an imbalance between the production of oxidants and antioxidant defenses; this generally occurs due to an excessive production of free radicals and/or the inefficiency of the antioxidant defense system [41]. Early life stress disrupts the limbic structures’ proper development and function, leading to lifelong susceptibility to stress, which affects behavior, cognition, and the reward system [42]. ELS has been extensively studied in the brain [43]; however, the effect on oxidative stress in organs has not yet been documented. In this work, we compared the enzyme responses involved in the oxidative stress pathway in rat pups subjected to MS under basal (basal control [BC] and basal MS [BMS]) or further stress conditions 3 h before euthanasia (stress control [SC] and stress MS [SMS]).

The first phase of the stress response (the sympathetic adrenal-medullar system) provides rapid physiological adaptation, resulting in short-term effects. The second phase involves a hormonal mechanism (hypothalamic pituitary adrenal axis-HPA), and its activation leads to short and long-term effects [44].

In the first phase, when a stressor is perceived by the brainstem, activation of the preganglionic autonomic neurons and hypophysiotropic neurons in the paraventricular nucleus of the hypothalamus (PVN) occurs, where the autonomic nervous system (ANS) generates the most immediate response to stressor exposure via its sympathetic and parasympathetic arms, and it provokes rapid alterations in physiological states through neural innervation to end organs, such as cardiovascular, respiratory, gastrointestinal, renal, endocrine, and other systems [43,45,46], as well as the HPA responses [43].

Physical stressors induce the activation of other brain structures that regulate the autonomic stress response, including PVN, the nucleus of the solitary tract (NTS), and the dorsomedial hypothalamus (DMH) [47]. The central noradrenergic system, specifically the locus coeruleus (LC), is involved in multiple neurochemical circuits and is connected to neuroanatomical structures involved in the stress response, such as the hippocampus, amygdala, and temporal neocortex [48].

Sympathetic system activation leads to signaling pathways that evoke changes in blood vessels, glands, visceral organs, and smooth muscles [44], and this occurs under conditions such as exercise and “fight-or-flight” reactions. The parasympathetic component of the ANS regulates the action and duration of autonomic responses, generating the so-called “vagal tone” of the cardiac and respiratory systems; moreover, it predominates during resting conditions [48,49].

The effect caused by any of these substances—acetylcholine, E, and NE—depends on the biochemical properties of the cells and the receptor distribution in a determined tissue [50]. E and NE interact with adrenergic receptors in the cell membranes of smooth muscles and numerous organs and neurons widespread in the CNS [44,50].

The main glucocorticoid in humans is cortisol, and its equivalent in rodents is corticosterone [51], both of which are associated with NE and ACTH [52]. They exert their effects on the brain by binding to two types of receptors: the glucocorticoid receptor (GR) and the mineralocorticoid receptor (MR). They modulate transcriptional factors in the nucleus, which increase mitochondrial membrane potential and mitochondria oxidation. Therefore, an increase in the cellular metabolic rate promotes ATP synthesis and spontaneous superoxide [3]. GR and MR characteristics, such as distribution, affinity, and mechanism of action, are determinants of homeostasis regulation under basal conditions or the promotion of adaptation through the stress response [43].

Glucocorticoids play a prominent role in controlling the stress response by regulating the HPA axis and the negative feedback on the hypothalamus and pituitary, affecting the secretion of CRH and ACTH, respectively [46]. Therefore, the continuous activation of the HPA axis induces oxidative stress [3,40] and an increase in ACTH and corticosterone responses to acute stress. [53].

When circulating catecholamine levels are elevated for prolonged periods, they can lead to different pathologies. Those pathologies may primarily affect the cardiovascular system (cardiac arrhythmias, angina, congestive heart failure, hypertension, and/or cardiac hypertrophy) [44,48,54].

E and NE can reduce oxidative stress by scavenging free radicals and sequestering metal ions [55]. Increased catecholamine levels are associated with an elevated production of ROS [56]. By regulating the nuclear factor, erythroid 2-related factor 2 (NRF2) likely contributes to antioxidant processes. NRF2 upregulates the enzymes involved in the cellular antioxidant response, such as glutathione disulfide, GPX, GST, thioredoxin, and thioredoxin reductase. For this reason, the upregulation of NRF2 has been considered key to governing the cellular antioxidant response [57].

When the techniques and values reported for the enzymes were considered, it was determined that our basal results are similar to the data on the following previous studies: SOD and CAT (Ewing J.F. et al., 1995; Jaiswal et al., 2013; Shakya et al., 2013 [27,58,59]), GPX (Bhanot R., 2019 [60]), GR (Bas H. 2021 [61]), GST (Jaiswal, 2013 [58]), NOS (Ajjuri, 2013 [62]; Rezaeizadeh A., 2011 [35]), and FRAP (Benzie I.F., 1996; Habibi E., 2018 [37,38]).

Superoxide dismutase (SOD), catalase (CAT), and glutathione peroxidase (GPX) are the most critical antioxidant enzymes playing key roles in redox homeostasis; they act in a mutually supportive defense action against ROS [63]. SOD enzymes catalyze the dismutation of the superoxide anion (O_2_^−^) to oxygen and hydrogen peroxide (H_2_O_2_). In our work, we observed increased enzymatic activity in almost all tissues in the SC compared with the BC, demonstrating the rapid neural response to acute stress explained above. In the MS groups, there was an increase in the activity of these enzymes, constituting an adaptation to chronic stress (14 days). There was an increase in oxidative stress due to stress exposure before euthanasia; in the case of basal conditions (BMS), the animals were separated from their mother and siblings for 3 h and for 14 days. This suggests an adaptation process, as few changes were observed in the enzymatic activities of the tissues; however, when the pups were subjected to further stress exposure (SMS), our findings showed either an increase or no change in those activities in most tissues.

Glucocorticoids increase alter the production of ROS, which induces several pathophysiological states, including myopathy, osteoporosis, diabetes, and hypertension [64]. In accordance with this, we observed an increase in the activity of these enzymes under the stress conditions (SC and SMS) studied.

In the SC, BMS and SMS groups, we observed an increase in GR activity in all organs, except for in the heart, where activity was decreased. An increase in GSH potentially counteracts ROS production [65]; thus, the increase in activity that we observed protects the organs against oxidative stress. The heart would probably be more exposed to diseases in adulthood [66].

An increase in corticosterone after MS has been reported [67,68]; it is likely that this chronic stress increases the transcription factor nuclear factor κB (NF-κB), which enhances the expression of antioxidant enzymes, such as SOD and GPX [63]. An increase in ROS generates oxidation in the cysteine thiols of a family of Ca^2+^ transporters, generating an efflux of Ca^2+^ into the cytoplasm, and this ion can directly activate antioxidant enzymes, such as GSH reductase and catalase [69,70].

Once H_2_O_2_ is formed, CAT or GPX will transform H_2_O_2_ into H_2_O+ O_2_. CAT is active when a high concentration of H_2_O_2_ is present, while GPX is active when H_2_O_2_ is low. The relationship between these two enzymes was more evident in the kidney and the adrenal glands. Their enzyme activities in the BMS groups increased, or no change was found in any of the tissues, except for in the heart. The enzyme activities in the SMS groups were significantly increased in the heart, liver, AGs, and pancreas.

Contrary to humans, the kidney continues to develop until postnatal day 14 in rats [71]; this could explain the differences observed between this organ and the other tissues studied. Chronic stress (MS) can permanently reduce or alter cell types, producing more resilient types or causing permanent deficits. Thus, a follow-up study in adult rats under the same conditions needs to be carried out.

Glutathione reductase (GR) catalyzes the reduction of glutathione disulfide (GSSG) to the sulfhydryl form of reduced glutathione (GSH), which is a critical molecule in resisting oxidative stress and maintaining the cell’s reducing environment [31]. We observed an increase in the enzyme activity of GR and GST in the SC and MS groups compared with in the BC groups, indicating a response to an increase in oxidative stress in the experimental groups.

This could be explained by the fact that catecholamine increases in response to stress, modulating the activity of the glutathione redox ratio (GSH/GSSG ratio), increasing MDA levels, and promoting the expression of oxidative stress enzyme genes [56].

Nitric oxide dilates blood vessels, raises blood supply, and lowers blood pressure. However, in organs, it helps to protect tissues from damage due to low blood supply. There was either no change or a reduction in BS, indicating a protective response from the organs. Under chronic stress conditions, a significant increase was observed in the heart and AGs, indicating that the oxidative stress in these organs was increased.

Besides the enzymatic activity described above, we tested two different parameters of oxidative stress: MDA and FRAP. First, malondialdehyde (MDA) is one of the final products of polyunsaturated fatty acid peroxidation in cells. An increase in free radicals causes the overproduction of MDA. The malondialdehyde level is commonly used as a marker of oxidative stress and antioxidant status. Higher amounts of lipoperoxidation (MDA) were found in the liver, kidney, and AGs in MS; this agrees with Gargan B.N., 2020, and Eskandari F, 2022 [56,68]. In the heart and pancreas, no reductions or differences in the groups were found.

The FRAP is an antioxidant capacity assay that measures the antioxidant potential in samples via the reduction of ferric iron (Fe^3+^) to ferrous iron (Fe^2+^) using the antioxidants present in the samples. Our study’s results suggest that exposure to acute stress reduces the antioxidant capacity in these organs, except for in the liver, where the capacity is increased, probably due to the organ’s functions. In the basal MS groups, the heart, liver, and kidney capacities were like those of the BC group, possibly due to a response to chronic stress. In the AGs, the FRAP values were increased. In the pancreas, they were decreased compared with the BC, perhaps due to the neural innervation previously explained or a different density of GC receptors in this tissue due to chronic stress. Finally, the SMS groups presented a further reduction in the values obtained, particularly in the heart, kidney, and AGs, but the liver and pancreas values were similar to those of the BMS groups. This suggests that the oxidative system in the tissues adapts after chronic stress conditions, as observed under basal conditions. However, the system was unable to respond to further exposure to stress, indicating that the antioxidant capacity was unable to eliminate ROS generation under this new stress condition.

We compared enzyme activities in the different organs across males and females; however, no statistical differences were obtained, perhaps because sexual maturation had not occurred by this postnatal day. In general, we observed similar patterns of enzyme activity in males and females of the same group. However, in terms of FRAP, an apparent different response was displayed in the hearts of the male and female rats in the SC group, suggesting differences in vulnerability between genders.

Study limitations: Although we present the results for different organs (the heart, liver, kidney, adrenal glands, and pancreas) in this work, we did not study other organs such as the lungs; thus, this remains to be studied. In this work, we used a homotypic stressor (same stressor) to evoke a stressful response in the control or MS animal groups, and, in future studies, we will investigate if the dynamic response to stress of enzyme activity changes in response to a heterotypic stressor in a similar fashion as it does to a homotypic stressor.

In general, all organs respond to stress using the first and second phases of the response described above. The differences found depend on the innervation of each organ and its functions. The increase in the enzymatic activities and oxidative parameters studied indicates an increase in oxidative stress associated with an increased risk of developing different chronic diseases and adverse physical and mental health outcomes later in life [1,46].

The DOHaD concept proposes that a “memory” of the early life environment is retained in later life [1]. Recently, it has been proposed that ELS can condition the methylation status within the methylated regions of several genes and contribute to numerous adverse health outcomes that manifest throughout the lifespan [72]. This morphological memory permanently alters the structure of specific organs or tissues, conditioning an impaired response to stress in the heart, liver, kidney, and pancreas.

The present results contribute to a better understanding of the mechanisms underlying the stress response in peripheral organs that carry a significant risk of developing a chronic disease in adulthood. Moreover, as previously stated, the windows of opportunities during early development may allow for the design of new drugs and treatments to modulate epigenetic processes, thereby improving or reversing deficits in adult health and generating new therapeutic strategies [73].

## 5. Conclusions

The integral response to acute stress in the organs of control pups generates a transient increased response in the antioxidant system. This is impaired by chronic stress, increasing ROS and altering their function.

## Figures and Tables

**Table 1:** Statistical analysis of superoxide dismutase (SOD) (**A**) and catalase (CAT) (**B**). Data represent the results of analysis of variance (ANOVA), carried out to determine significant differences. F and *p* values are presented. N.S: Non-significant. Regarding factor 1, treatment, BC, SC, BMS, and SMS groups showed significant differences. Regarding factor 2, euthanasia protocol, there were no significant differences for some tissues and gender. Regarding factor 3, gender, there were no significant differences in any of the organs. Interaction between 1 × 2 factors; Regarding SOD, there was no significance in the male liver. Regarding CAT, 0 no significance was obtained for the female pancreas.

**A**				
**SOD**				
	Factor 1	Factor 2	Factor 3	
	Treatment	Euthanasia	Gender	Interaction
**Heart**				
Male	F (1, 76) = 170.7, *p* < 0.0001	F (1, 76) = 64.51, *p* < 0.0001	NS	F (1, 76) = 215.8, *p* < 0.0001
Female	F (1, 68) = 314.0, *p* < 0.0001	NS	NS	F (1, 68) = 273.8, *p* < 0.0001
**Liver**				
Male	F (1, 64) = 160.3, *p* < 0.0001	NS	NS	NS
Female	F (1, 54) = 116.5, *p* < 0.0001	F (1, 54) = 26.10, *p* < 0.0001	NS	F (1, 54) = 104.9, *p* < 0.0001
**Kidney**				
Male	F (1, 64) = 448.2, *p* < 0.0001	F (1, 64) = 152.3, *p* < 0.0001	NS	F (1, 64) = 775.3, *p* < 0.0001
Female	F (1, 54) = 628.0, *p* < 0.0001	F (1, 54) = 470.2, *p* < 0.0001	NS	F (1, 54) = 148.0, *p* < 0.0001
**Adrenal Gland**				
Male	F (1, 76) = 1222, *p* < 0.0001	F (1, 76) = 2842, *p* < 0.0001	NS	F (1, 76) = 24.84, *p* < 0.0001
Female	F (1, 68) = 27.49, *p* < 0.0001	F (1, 68) = 2219, *p* < 0.0001	NS	F (1, 68) = 73.57, *p* < 0.0001
**Pancreas**				
Male	F (1, 76) = 424.0, *p* < 0.0001	NS	NS	F (1, 76) = 4.275, *p* = 0.0421
Female	F (1, 68) = 108.0, *p* < 0.0001	NS	NS	F (1, 68) = 8.316, *p* = 0.0053
**B**				
**CAT**				
	Factor 1	Factor 2	Factor 3	
	Treatment	Euthanasia	Gender	Interaction
**Heart**				
Male	F (1, 76) = 158.7, *p* < 0.0001	F (1, 76) = 406.2, *p* < 0.0001	NS	F (1, 76) = 75.63, *p* < 0.0001
Female	F (1, 68) = 32.76, *p* < 0.0001	F (1, 68) = 617.0, *p* < 0.0001	NS	F (1, 68) = 12.77, *p* < 0.0007
**Liver**				
Male	F (1, 32) = 19.30, *p* = 0.0001	NS	NS	F (1, 32) = 18.29, *p* = 0.0002
Female	F (1, 40) = 6.978, *p* = 0.0117	F (1, 40) = 6.377, *p* = 0.0156	NS	F (1, 40) = 2.315, *p* = 0.1360
**Kidney**				
Male	F (1, 64) = 314.2, *p* < 0.0001	F (1, 64) = 27.75, *p* < 0.0001	NS	F (1, 64) = 57.50, *p* < 0.0001
Female	F (1, 54) = 362.1, *p* < 0.0001	NS	NS	F (1, 54) = 132.0, *p* < 0.0001
**Adrenal Gland**				
Male	F (1, 76) = 37.37, *p* < 0.0001	F (1, 76) = 460.0, *p* < 0.0001	NS	F (1, 76) = 360.7, *p* < 0.0001
Female	F (1, 68) = 8.168, *p* = 0.0057	F (1, 68) = 855.5, *p* < 0.0001	NS	F (1, 68) = 9.710, *p* = 0.0027
**Pancreas**				
Male	F (1, 76) = 63.12, *p* < 0.0001	F (1, 76) = 157.1, *p* < 0.0001	NS	F (1, 76) = 20.70, *p* < 0.0001
Female	F (1, 68) = 76.72, *p* < 0.0001	F (1, 68) = 35.77, *p* < 0.0001	NS	NS

**Table 2:** Statistical analysis of glutathione peroxidase (GPX) (**A**) and glutathione reductase (GR) (**B**). Data represent the results of the analysis of variance (ANOVA), carried out to determine significant differences. F and *p* values are presented. N.S. non-significant. The first factor was the treatment of the groups: BC, SC, BMS, and SMS. There was a significant difference in GPX and GR in all organs. The second factor was euthanasia. Most of the organs presented significant differences in GPX and GR, except for the pancreas in GPX. The interaction (Factor 1 × 2) presented significant differences in most of the organs. The third factor, gender, showed no significant differences in GPX or GR in all organs.

**A**				
**GPX**				
	Factor 1	Factor 2	Factor 3	
	Treatment	Euthanasia	Gender	Interaction
**Heart**				
Male	F (1, 76) = 745.4, *p* < 0.0001	F (1, 76) = 304.2, *p* < 0.0001	NS	F (1, 76) = 841.4, *p* < 0.0001
Female	F (1, 68) = 81.53, *p* < 0.0001	NS	NS	F (1, 68) = 194.9, *p* < 0.0001
**Liver**				
Male	NS	F (1, 28) = 44.03, *p* < 0.0001	NS	F (1, 28) = 26.69, *p* < 0.0001
Female	F (1, 33) = 20.07, *p* < 0.0001	F (1, 33) = 97.54, *p* < 0.0001	NS	F (1, 33) = 6.239, *p* = 0.0177
**Kidney**				
Male	F (1, 64) = 197.2, *p* < 0.0001	F (1, 64) = 103.0, *p* < 0.0001	NS	F (1, 64) = 114.8, *p* < 0.0001
Female	F (1, 54) = 110.9, *p* < 0.0001	F (1, 54) = 77.42, *p* < 0.0001	NS	F (1, 54) = 101.6, *p* < 0.0001
**Adrenal Gland**				
Male	F (1, 76) = 43.24, *p* < 0.0001	F (1, 76) = 216.7, *p* < 0.0001	NS	F (1, 76) = 36.39, *p* < 0.0001
Female	F (1, 68) = 97.29, *p* < 0.0001	F (1, 68) = 226.7, *p* < 0.0001	NS	F (1, 68) = 115.7, *p* < 0.0001
**Pancreas**				
Male	F (1, 76) = 152.8, *p* < 0.0001	NS	NS	F (1, 76) = 142.5, *p* < 0.0001
Female	F (1, 68) = 81.43, *p* < 0.0001	NS	NS	F (1, 68) = 59.27, *p* < 0.0001
**B**				
**GR**				
	Factor 1	Factor 2	Factor 3	
	Treatment	Euthanasia	Gender	Interaction
**Heart**				
Male	F (1, 76) = 38.44, *p* < 0.0001	F (1, 76) = 35.57, *p* < 0.0001	NS	F (1, 76) = 121.9, *p* < 0.0001
Female	F (1, 68) = 19.90, *p* < 0.0001	F (1, 68) = 22.54, *p* < 0.0001	NS	NS
**Liver**				
Male	F (1, 74) = 274.1, *p* < 0.0001	F (1, 74) = 7.397, *p* = 0.0081	NS	F (1, 74) = 19.47, *p* < 0.0001
Female	F (1, 66) = 301.4, *p* < 0.0001	F (1, 66) = 4.934, *p* = 0.0298	NS	F (1, 66) = 64.92, *p* < 0.0001
**Kidney**				
Male	F (1, 64) = 193.0, *p* < 0.0001	F (1, 64) = 181.4, *p* < 0.0001	NS	F (1, 64) = 199.1, *p* < 0.0001
Female	F (1, 54) = 9.826, *p* = 0.0028	F (1, 54) = 38.73, *p* < 0.0001	NS	F (1, 54) = 30.48, *p* < 0.0001
**Adrenal Gland**				
Male	F (1, 76) = 2889, *p* < 0.0001	F (1, 76) = 336.7, *p* < 0.0001	NS	F (1, 76) = 237.7, *p* < 0.0001
Female	F (1, 68) = 1561, *p* < 0.0001	F (1, 68) = 71.25, *p* < 0.0001	NS	F (1, 68) = 146.5, *p* < 0.0001
**Pancreas**				
Male	F (1, 76) = 2.902, *p* = 0.0925	F (1, 76) = 75.19, *p* < 0.0001	NS	F (1, 76) = 16.26, *p* = 0.0001
Female	F (1, 68) = 15.81, *p* = 0.0002	F (1, 68) = 73.68, *p* < 0.0001	NS	F (1, 68) = 74.69, *p* < 0.0001

**Table 3:** Statistical analysis of glutathione S transferase (GST) (**A**) and nitric oxide synthase (NOS) (**B**). Data represent results of the analysis of variance (ANOVA) carried out to determine significant differences. F and *p* values are presented. N.S. non-significant. Regarding factor A, treatments, BC, SC, BMS, and SMS groups showed significant differences in heart, kidney, AGs, and pancreas; in liver, no significant differences were found in GST; in heart, kidney, and pancreas, there were significant differences, while, in liver and AGs, there were no significant differences. Regarding factor 2, euthanasia, no significant differences were found for GST and NOS in AGs. The interaction between factor AXB showed no significant differences in GST in AGs and pancreas, or in NOS in heart and AGs. Regarding factor 3, gender, there were no significant differences in any of the organs.

**A**				
**GST**				
	Factor 1	Factor 2	Factor 3	
	Treatment	Euthanasia	Gender	Interaction
**Heart**				
Male	F (1, 76) = 13.93, *p* = 0.0004	F (1, 76) = 858.0, *p* < 0.0001	NS	F (1, 76) = 14.01, *p* = 0.0004
Female	F (1, 68) = 67.55, *p* < 0.0001	F (1, 68) = 465.3, *p* < 0.0001	NS	F (1, 68) = 67.71, *p* < 0.0001
**Liver**				
Male	NS	F (1, 76) = 292.1, *p* < 0.0001	NS	F (1, 76) = 6.485, *p* = 0.0129
Female	NS	F (1, 68) = 312.7, *p* < 0.0001	NS	F (1, 68) = 26.43, *p* < 0.0001
**Kidney**				
Male	F (1, 64) = 8.369, *p* = 0.0052	F (1, 64) = 23.44, *p* < 0.0001	NS	NS
Female	F (1, 54) = 17.88, *p* < 0.0001	F (1, 54) = 32.87, *p* < 0.0001	NS	F (1, 54) = 11.79, *p* = 0.0011
**Adrenal Gland**				
Male	F (1, 76) = 49.68, *p* < 0.0001	NS	NS	NS
Female	F (1, 68) = 14.07, *p* = 0.0004	NS	NS	NS
**Pancreas**				
Male	F (1, 76) = 9.529, *p* = 0.0028	F (1, 76) = 93.09, *p* < 0.0001	NS	NS
Female	F (1, 68) = 14.88, *p* = 0.0003	F (1, 68) = 91.27, *p* < 0.0001	NS	NS
**B**				
**NOS**				
	Factor 1	Factor 2	Factor 3	
	Treatment	Euthanasia	Gender	Interaction
**Heart**				
Male	F (1, 74) = 14.31, *p* = 0.0003	F (1, 74) = 63.35, *p* < 0.0001	NS	NS
Female	F (1, 66) = 9.204, *p* = 0.0035	F (1, 66) = 53.84, *p* < 0.0001	NS	NS
**Liver**				
Male	NS	F (1, 74) = 40.64, *p* < 0.0001	NS	F (1, 74) = 353.3, *p* < 0.0001
Female	NS	NS	NS	NS
**Kidney**				
Male	F (1, 64) = 311.1, *p* < 0.0001	F (1, 64) = 275.9, *p* < 0.0001	NS	F (1, 64) = 71.66, *p* < 0.0001
Female	NS	F (1, 54) = 102.2, *p* < 0.0001	NS	NS
**Adrenal Gland**				
Male	NS	NS	NS	NS
Female	NS	NS	NS	NS
**Pancreas**				
Male	F (1, 76) = 370.1, *p* < 0.0001	F (1, 76) = 285.4, *p* < 0.0001	NS	F (1, 76) = 90.65, *p* < 0.0001
Female	F (1, 68) = 5.207, *p* = 0.0256	F (1, 68) = 71.21, *p* < 0.0001	NS	NS

**Table 4:** Statistical analysis of malondialdehyde (MDA) (**A**) and ferric-reducing ability potential (FRAP). (**B**). Data represent the results of the analysis of variance (ANOVA), carried out to determine significant differences. F and *p* values are presented. N.S. Regarding factor 1 (treatment), factor 2 (euthanasia protocol), and the interaction between 1 × 2 factors, significant differences were observed in MDA and FRAP in all organs. Regarding factor 3 (gender), no significant differences were observed.

**A**				
**MDA**				
	Factor 1	Factor 2	Factor 3	
	Treatment	Euthanasia	Gender	Interaction
**Heart**				
Male	F (1, 76) = 98.47, *p* < 0.0001	F (1, 76) = 594.5, *p* < 0.0001	NS	F (1, 76) = 61.47, *p* < 0.0001
Female	F (1, 68) = 40.28, *p* < 0.0001	F (1, 68) = 383.7, *p* < 0.0001	NS	F (1, 68) = 91.20, *p* < 0.0001
**Liver**				
Male	F (1, 76) = 54.75, *p* < 0.0001	F (1, 76) = 413.4, *p* < 0.0001	NS	F (1, 76) = 54.56, *p* < 0.0001
Female	F (1, 68) = 20.19, *p* < 0.0001	F (1, 68) = 126.1, *p* < 0.0001	NS	F (1, 68) = 160.5, *p* < 0.0001
**Kidney**				
Male	F (1, 64) = 100.0, *p* < 0.0001	F (1, 64) = 1036, *p* < 0.0001	NS	NS
Female	F (1, 54) = 157.3, *p* < 0.0001	F (1, 54) = 570.7, *p* < 0.0001	NS	F (1, 54) = 37.56, *p* < 0.0001
**Adrenal Gland**				
Male	F (1, 76) = 300.1, *p* < 0.0001	F (1, 76) = 5804, *p* < 0.0001	NS	F (1, 76) = 26.93, *p* < 0.0001
Female	F (1, 68) = 439.3, *p* < 0.0001	F (1, 68) = 5699, *p* < 0.0001	NS	F (1, 68) = 12.70, *p* = 0.0007
**Pancreas**				
Male	F (1, 76) = 21.60, *p* < 0.0001	F (1, 76) = 1690, *p* < 0.0001	NS	F (1, 76) = 57.87, *p* < 0.0001
Female	F (1, 68) = 156.9, *p* < 0.0001	F (1, 68) = 647.1, *p* < 0.0001	NS	F (1, 68) = 18.52, *p* < 0.0001
**B**				
**FRAP**				
	Factor 1	Factor 2	Factor 3	
	Treatment	Euthanasia	Gender	Interaction
**Heart**				
Male	F (1, 76) = 237.1, *p* < 0.0001	F (1, 76) = 106.0, *p* < 0.0001	NS	F (1, 76) = 96.81, *p* < 0.0001
Female	F (1, 68) = 22.77, *p* < 0.0001	F (1, 68) = 269.5, *p* < 0.0001	NS	F (1, 68) = 175.5, *p* < 0.0001
**Liver**				
Male	F (1, 76) = 235.1, *p* < 0.0001	F (1, 76) = 165.0, *p* < 0.0001	NS	F (1, 76) = 150.5, *p* < 0.0001
Female	F (1, 68) = 36.95, *p* < 0.0001	F (1, 68) = 55.07, *p* < 0.0001	NS	F (1, 68) = 76.85, *p* < 0.0001
**Kidney**				
Male	F (1, 64) = 181.9, *p* < 0.0001	F (1, 64) = 115.2, *p* < 0.0001	NS	F (1, 64) = 6.096, *p* = 0.0162
Female	F (1, 54) = 8.938, *p* = 0.0042	F (1, 54) = 9.625, *p* = 0.0031	NS	NS
**Adrenal Gland**				
Male	F (1, 76) = 226.4, *p* < 0.0001	F (1, 76) = 390.8, *p* < 0.0001	NS	F (1, 76) = 134.7, *p* < 0.0001
Female	F (1, 68) = 21.24, *p* < 0.0001	F (1, 68) = 55.94, *p* < 0.0001	NS	F (1, 68) = 56.44, *p* < 0.0001
**Pancreas**				
Male	F (1, 76) = 15.82, *p* = 0.0002	F (1, 76) = 158.0, *p* < 0.0001	NS	F (1, 76) = 75.20, *p* < 0.0001
Female	NS	F (1, 68) = 47.23, *p* < 0.0001	NS	F (1, 68) = 29.28, *p* < 0.0001

## Data Availability

The original data presented in the study are openly available at: https://docs.google.com/spreadsheets/d/1SHmd92kZiezRX-QTlRQhzXf8YxC5MbCw/edit?usp=drive_link&ouid=104513107905361347180&rtpof=true&sd=true, accessed on 27 June 2024.
